# Management of uterine sarcomas and prognostic indicators: real world data from a single-institution

**DOI:** 10.1186/s12885-018-5156-1

**Published:** 2018-12-13

**Authors:** Anastasios Kyriazoglou, Michael Liontos, Dimitrios C Ziogas, Flora Zagouri, Kostantinos Koutsoukos, Giorgos Tsironis, Anna Tsiara, Maria Kaparelou, Roubini Zakopoulou, Nikolaos Thomakos, Dimitrios Haidopoulos, Irene Papaspyrou, Alexandros Rodolakis, Aristotelis Bamias, Meletios Athanasios Dimopoulos

**Affiliations:** 1 Department of Clinical Therapeutics, Oncology Unit, Alexandra Hospital, Vasilisis Sofias, 80 Athens, Greece; 2 Obstetrics and Gynecology Department, Alexandra Hospital, Vasilisis Sofias, 80 Athens, Greece; 3 Pathology Department, Alexandra Hospital, Vasilisis Sofias, 80 Athens, Greece

**Keywords:** Uterine sarcomas, Prognostic factors, Mitotic index

## Abstract

**Background:**

Uterine sarcomas consist a heterogeneous group of mesenchymal gynecological malignancies with unclear therapeutic recommendations and unspecific but poor prognosis, since they usually metastasize and tend to recur very often, even in early stages.

**Methods:**

We retrospectively analyzed all female patients with uterine sarcomas treated in our institution over the last 17 years. Clinico-pathological data, treatments and outcomes were recorded. Kaplan-Meier curves were plotted and time-to-event analyses were estimated using Cox regression.

**Results:**

Data were retrieved from 61 women with a median age of 53 (range: 27–78) years, at diagnosis. Fifty-one patients were diagnosed with leiomyosarcoma (LMS), 3 with high grade endometrial stromal sarcoma (ESS), 5 with undifferentiated uterine sarcoma (UUS), 1 with Ewing sarcoma (ES) and 1 with Rhabdomyosarcoma (RS). 24 cases had stage I, 7 stage II, 14 stage III and 16 stage IV disease. Median disease-free survival (DFS) in adjuvant approach was 18.83 months, and median overall survival (OS) 31.07 months. High mitotic count (> 15 mitoses) was significantly associated with worse OS (*P* < 0.001) and worse DFS (*P* = 0.028).

**Conclusions:**

Mitotic count appears to be independent prognostic factor while further insights are needed to improve adjuvant and palliative treatment of uterine sarcomas.

## Background

Sarcomas form a heterogeneous group of malignant tumors of mesenchymal origin. Occasionally these tumors may originate from the uterus (uterine sarcomas) including mainly leiomyosarcomas (LMS), endometrial stromal sarcomas (ESS) and undifferentiated uterine sarcomas (UUS), according to the College of American Pathologists’ classification for uterine sarcomas (Table 1) [1]. Uterine sarcomas account for 3–7% of all uterine cancers and affect women of all ages with higher incidence between 5th to 7th decades of life [2]. The prognosis of these tumors remains poor, with 5-year survival rate reaching 40%. Further insights are needed in order to predict the course of uterine sarcomas and improve their treatment. Up to now, several characteristics of uterine sarcomas are identified as prognostic factors including tumor grading, FIGO staging (International Federation of Oncology and Obstetrics), mitotic count, age and necrosis [3–5]. The French Federation of Anticancer Centers (FNCLCC) has developed a scoring system for grading of soft tissue sarcomas, evaluating 3 histologic criteria: tumor differentiation, mitotic count and necrosis [6]. However, its use has not been generalized as a prognostic tool for uterine sarcomas [7]. Apart from their common origin, these tumors present with distinct biological and molecular profiles that may also determine their behavior under treatment [8]. Several different pathways with oncogenic importance are implicated in the evolution of these sarcomas [9]. For instance, LMSs harbor complex karyotypes and genetic alterations with gains and losses of several genetic loci [10], but lack genetic changes within specific genes. In addition, ESSs are characterized by the existence of specific fusion genes a) YWHAE-FAM22 and ZC3H7B-BCOR for high-grade ESS [11, 12] or b) JAZF1 rearrangements and PHF1 rearrangements for low-grade ESS [13]. On the contrary, UUSs demonstrate complex genetic alterations totally distinct from the other two histological subsets [13].

**Table 1:** Classification system for uterine Sarcomas, College of the American Pathologists

College of American Pathologists classification system for uterine sarcomas	
Histologic type (more than one may apply)	
Leiomyosarcoma	
Low-grade endometrial stromal sarcoma*	
Low-grade endometrial stromal sarcome with:	
Smooth muscle differentiation	
Sex cord elements	
Glandular elements	
Other (specify)	
High-grade endometrial stromal sarcoma	
Undifferentiated uterine/ endometrial sarcoma	
Adenosarcoma	
Adenosarcoma with:	
Rhabdomyoblastic differentiation	
Cartilaginous differentiation	
Osseous differentiation	
Other heterologous element (specify)	
Adenosarcoma with sarcomatous overgrowth	
Other (specify)	

*Low-grade endometrial sarcoma is distinguished from benign endometrial stromal nodule by infiltration into the surrounding myometrium and/or lympovascular invasion. Minor marginal irregularity in the form of tongues < 3 mm(up to three) is allowable for an endometrial stromal nodule. This protocol does not apply to endometrial

The therapeutic approach of uterine sarcomas is similar to the rest of soft tissue sarcomas [14, 15]. Surgery remains the mainstay of therapy but recurrence rates in operable disease (stages I-ΙΙΙ) are high [16]. The role of adjuvant therapy to those women is still a matter of debate with controversial results by small studies [16–21]. Thus, adjuvant chemotherapy for uterine sarcomas is under consideration with low level of evidence in the existing guidelines [15, 22]. Despite the recent advances on the treatment of metastatic or unresectable sarcoma with the addition of olaratumab (a human antiplatelet derived growth factor receptor-α monoclonal antibody) to doxorubicin [15, 22] and the introduction of eribulin in patients with advanced LMS [23], the conventional adriamycin-based chemotherapy remains the gold therapeutic standard in advanced setting of the disease [14]. Several other chemotherapeutic agents including trabectedin and pazopanib have also been investigated but without significant survival benefits [24, 25] and the prognosis of women with metastatic disease remaining dismal with 2-year survival roughly reaching 30%.

Under this perspective, we reviewed the medical files of diagnosed patients in our institution during the last 15 years and retrospectively analyzed their clinicopathological characteristics in order to recognize parameters that affect their prognosis.

## Methods

### Selection of patients

We retrospectively analyzed all female patients with uterine sarcomas treated in our institution from 2001 to 2016. All included patients in our analysis had histological diagnosis of uterine sarcoma and had undergone staging of their disease. The ethics committee of the Hospital approved the study and patients have signed informed consent for the analysis of their data.

### Data collection-definition of survival times

For each patient, the following data were collected: i) clinicopathological characteristics of sarcoma patients at the time of diagnosis including age, sex, PS (performance status), histologic subtype, grade, stage, mitotic count; ii) local and systemic therapies received, such as the type of surgery, adjuvant or 1st-line chemotherapy for metastatic or recurrent disease and later regimens, the use of radiotherapy; as well as iii) the clinical outcomes including disease progression or death, the site of recurrence/metastasis and the times of overall survival (OS), progression free survival (PFS), disease free survival (DFS). OS was defined as the time period from the date of diagnosis of gynecological sarcoma to the date of the last follow-up or death and PFS was defined as the time during and after the primary treatment (surgery and adjuvant or 1st line) with no clinical or imaging signs of sarcoma relapse/progression. RFS (recurrence free survival) was defined as the time to recurrence after the adjuvant treatment. Data completeness exceeded 95%.

### Statistical analysis

For categorical variables, data are presented as frequencies with their corresponding 95% confidence intervals (95%CIs), and for continuous variables as median with observed range (minimum–maximum). The 95% CIs of proportions were computed using the modified Wald method. To compare categorical variables, we used the Chi square test or Fischer’s exact test where appropriate. To compare continuous variables, the Mann–Whitney (two-tailed) test was used. Survival curves were plotted and time-to-event analyses were estimated using the Kaplan-Meier method; differences between curves were analyzed using the log-rank test. The median follow-up times were computed using the reverse Kaplan-Meier method. Unadjusted and adjusted hazard ratios (HR) with the respective 95% CIs were estimated using univariate and multivariate Cox regression analysis, respectively. The multivariate Cox regression analysis examined the effect on OS and PFS after adjustment for all already known prognostic parameters at baseline. These baseline parameters were included in the final multi-regression analysis as dichotomous variables as following: (1) age, using 65 years as elderly cutoff (> 65 years =1, ≤65 years =0); (2) advanced disease (tumor stage III or IV = 1, tumor stage = I or II = 0); (3) tumor size, using 10 cm as cutoff (> 10 cm = 1, ≤10 cm = 0); (4) grading (grade 3 = 1, grade 1 or 2 = 0); (5) mitotic index, using the median value of mitotic index of our cohort as the dichotomous threshold (high mitotic index = 1, low mitotic index = 0). Moreover, unadjusted and adjusted odds ratios (OR) with the respective 95% CIs were estimated using logistic regression in order to examine the effect of baseline parameters on the events of death and progression without taking into account the time effect. Statistical analyses were performed using SPSS software package, version 21 (Computing Resource Centre, Santa Monica, California, USA) and GraphPad Prism software (GraphPad Software Inc., La Jolla, California, USA). Statistical significance was defined as a *P*-value of less than 0.05 for all comparisons.

## Results

### Baseline characteristics

From 2001 to 2016, 61 consecutive cases of uterine sarcomas treated in our Department, were included in the retrospective analysis. Table 2 summarizes their clinicopathological characteristics. The median age was 53 (range: 27–78) years with the majority of them (85.25%) being younger than 65 years old. Almost half of the patients (30 cases, 49.18%) were diagnosed with advanced disease; 14 cases (22.95%) had stage III disease and 16 (26.23%) had metastatic disease (stage IV). Patients with de novo stage IV disease had metastases in several sites including lungs (10 cases), liver (4 cases), peritoneal/retroperitoneal depositions (3 cases), bones (3 cases) and bladder (1 case). According to the histological evaluation of their biopsies, 51 cases (83.61%) were diagnosed with LMS, 3 cases (4.92%) with high grade ESS, 5 cases (8.20%) with UUS, 1 case (1.64%) with Ewing’s sarcoma (ES) and 1 case (1.64%) with rhabdomyosarcoma (RS). The median tumor size was 14 (range: 4–30) centimeters (cm) and 45 of 61 patients (73.77%) were diagnosed with a primary lesion larger than 10 cm. In the majority of cases (81.97%) sarcoma presented with cellular differentiation grade 3, while the calculated mitotic index had a median value of 15 (range: 4–54) mitoses for every 10 high power fields (HPF). More than 15 mitoses per 10HPF were detected in 22 cases (36.07%).

**Table 2:** Clinicopathological baseline characteristics of included patients with gynecological uterine sarcomas

Number of patients	61
Age (years)	
Median	53
below 65	52 (85.25%)
over 65	9 (14.75%)
Histology	
LMS	51 (83.61%)
ESS	3 (4.92%)
UUS	5 (8.20%)
ES	1 (1.64%)
RS	1 (1.64%)
Tumor size	
< 10 cm	16 (26.23%)
> 10 cm	45 (73.77%)
Grade	
Grade 1	3 (4.92%)
Grade 2	6 (9.84%)
Grade 3	50 (81.97%)
FIGO Stage	
Stage I	24 (39.34%)
Stage II	7 (18.03%)
Stage III	14(22.95%)
Stage IV	16 (26.23%)
Mitotic index	
Mitosis < 15 per HPF	25/47 (53.19%)
Mitosis > 15 per HPF	22/47 (46.81%)

Abbreviations: *LMS* Leiomyosarcoma, *ESS* Endometrial stromal sarcoma, *UUS* Undifferentiated uterine sarcoma, *FIGO* International Federation of Oncology and Obstetrics, *HPF* High power fields

### Primary treatment

The majority of our patients (59 of 61 patients, 96.72%, underwent bilateral salpingo-ophorectomy including 15 patients with already known metastatic disease. In the later cases, the aim of the operation was palliative either to alleviate abdominal discomfortness or to control uterine bleeding. The two cases that did not undergo surgery, already advanced disease (stage IV) was confirmed histologically by laparoscopic biopsies. Based on their postoperative CT scans, 42 women (68.85%) were considered free of residual disease and received adjuvant chemotherapy. Among them, twenty-nine patients (70.73%) received either doxorubicin or doxorubicin-based regimens and 8 (19.51%) received gemcitabine and docetaxel as adjuvant chemotherapy. Four patients (9.76%) received other regimens, mainly platinum-based. Fourteen patients received adjuvant radiotherapy to the pelvis (11 patients with stages I to III, and 3 patients with stage IV disease for palliative intent). Adjuvant RT offered no significant survival benefit in the univariate analysis and it was not incorporated into our multivariate analysis due to small sample size in the subgroups. Median duration of adjuvant chemotherapy was 3.7 (0.93–13.90) months and did not differ significantly according to the type of regimen; 3.53 months for anthracycline-based regiments vs 3.7 months for the gemcitabine/docetaxel doublet. Among patients that received adjuvant chemotherapy, median disease-free survival (DFS) was 18.83 (range: 2.53–129.27) months. Thirty patients relapsed after adjuvant chemotherapy, nine with local only disease and 21 with distant disease involving lung as the most common site of relapse. Pattern of relapse was not related to DFS.

### Treatment of metastatic disease

Thirty-nine patients were treated for metastatic disease: 14 had metastases at diagnosis, while 25 relapsed after surgery (24 also had adjuvant chemotherapy).

**Fig. 1:**
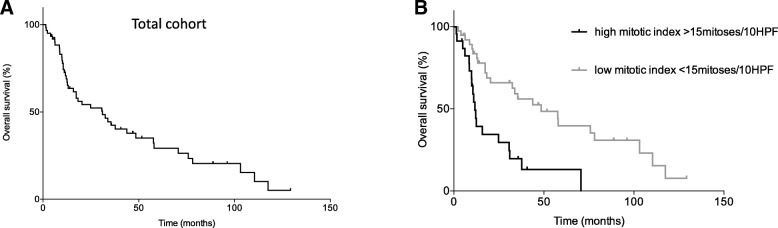
**a** Overall survival in months, **b** Time to event analysis, Overall survival in months in correlation with mitotic index, high mitotic index: > 15 mitoses/10HPF, low mitotic index: < 15 mitoses/10HPF

Up to October 2017, 41 patients experienced progression or relapse of their sarcoma (67.21%), while 43 had died (70.49%). For the whole cohort of patients, median OS was 31.07 months (range: 1.43–129.27 months) (Fig. 1a) and median PFS was 5.03 (range: 0.3–49.97) months. Among de novo metastatic patients mPFS was 5.17 (range: 0.93–15.40) months and for those received prior adjuvant treatment 5.0 (range: 1.23–49.97) months.

### Baseline prognostic factors

The effect of baseline dichotomized parameters including age at the time of diagnosis, tumor size, histological subtype, grade, mitotic index and initial stage of the disease in the prognosis of OS was examined in univariate and multivariate setting. In time-to-event analysis, the high mitotic index was significantly associated with worse OS (log-rank *p* = 0.0002, HR = 3.441, 95%CI: 1.649–7.181) (Fig. 1b) (Table 3). In the multivariate analysis, after adjustment of all baseline parameters, the high mitotic index (> 15mitoses/10HPF) retained its statistically prognostic significance for OS (adjusted HR = 3.283, 95%CI: 1.426–7.559, *p* = 0.005). In order to predict any DFS benefit, all above aforementioned parameters as well as the type of adjuvant chemotherapy were also re-examined. In addition to high mitotic index (HR = 2.687, 95%CI: 1.160–6.471, *p* = 0.028), the grade 3 differentiation of the sarcoma (HR = 3.426, 95%CI: 1.014–11.569, *p* = 0.047) was also recognized to be associated with worse DFS in the univariate setting (Table 3). However, in the multivariate setting, grade 3 differentiation did not reach to statistical significance, while large tumor size (> 10 cm) (adjusted HR = 4.071, 95%CI: 1.205–13.752, *p* = 0.024) was added to the baseline high mitotic index (adjusted HR = 3.041, 95%CI: 1.127–8.204, p = 0.028) as important predictors for DFS (Table 3). Trying to recognize the responders based on their baseline characteristics, no significant differences were found between patients that relapsed and not relapsed after their adjuvant approach. It is important that all identified parameters describe the strong effect of initial cellular behavior in the outcome of sarcoma.

**Table 3:** Estimated effects of prognostic parameters at the diagnosis of gynecological sarcoma in patients’ outcomes

Parameters	OS			
	Total number of patients = 61			
	HR (95%CI)	*P*-value	Adjusted HR (95%CI)	*P*-value
Elderly age (> 65 vs. ≤65 years)	1.564 (0.690–3.545)	0.284	1.376 (0.583–2.764)	0.466
Advanced disease (stage III/IV vs. I/II)	1.700 (0.982–3.114)	0.086	1.300 (0.641–2.636)	0.466
Tumor size (> 10 cm vs. ≤10 cm)	1.418 (0.691–2.909)	0.341	1.403 (0.586–3.356)	0.447
Grade (3 vs. 2/1)	2.144 (0.920–4.996)	0.077	1.758 (0.625–4.945)	0.285
Histological subtype (LMS vs. Others)	0.968 (0.373–2.512)	0.947	0.989 (0.362–2.699)	0.982
Mitotic index (>15mitoses/10HPF vs. ≤15mitoses/10HPF)	3.441 (1.649–7.181)	0.001	3.283 (1.426–7.559)	0.005
	DFS			
	Total number of patients = 42			
	HR (95%CI)	P-value	Adjusted HR (95%CI)	P-value
Elderly age (> 65 vs. ≤65 years)	1.404 (0.413–4.773)	0.586	0.671 (0.138–3.266)	0.621
Advanced disease (stage III/IV vs. I/II)	1.267 (0.586–2.738)	0.547	0.970 (0.352–2.676)	0.953
Tumor size (> 10 cm vs. ≤10 cm)	2.113 (0.853–5.235)	0.106	4.071 (1.205–13.752)	0.024
Grade (3 vs. 2/1)	3.426 (1.014–11.569)	0.047	4.121 (0.927–18.326)	0.063
Histological subtype (LMS vs. Others)	0.662 (0.263–1.667)	0.381	1.414 (0.410–4.874)	0.583
Mitotic index (>15mitoses/10HPF vs. ≤15mitoses/10HPF)	2.687 (1.160–6.471)	0.028	3.041 (1.127–8.204)	0.028
Adjuvant regimen (Antracycline-based vs. other chemotherapy)	0.783 (0.354–1.731)	0.546	0.555 (0.172–1.794)	0.325
	1st line PFS			
	Total number of patients = 38			
	HR (95%CI)	P-value	Adjusted HR (95%CI)	P-value
Elderly age (> 65 vs. ≤65 years)	1.300 (0.493–3.433)	0.596	0.927 (0.230–3.728)	0.915
Tumor size (> 10 cm vs. ≤10 cm)	0.679 (0.303–1.522)	0.347	0.898 (0.270–2.986)	0.861
Grade (3 vs. 2/1)	0.471 (0.188–1.181)	0.108	0.220 (0.047–1.032)	0.055
Histological subtype (LMS vs. Others)	0.662 (0.263–1.667)	0.381	0.333 (0.087–1.282)	0.110
Mitotic index (>15mitoses/10HPF vs. ≤15mitoses/10HPF)	1.158 (0.512–2.621)	0.752	1.463 (0.454–4.721)	0.524
Adjuvant chemotherapy (Yes vs. no)	1.324 (0.635–2.762)	0.454	1.066 (0.337–3.375)	0.914

Abbreviations: *OS* Overall survival, *PFS* Progression-free survival, *HPF* High power fields, *OR* Odd ratio, *HR* Hazard ratio, 95%*CI* 95% confidence intervals

## Discussion

Uterine sarcomas are rare tumors with highly malignant behavior [2]. They tend to metastasize and recur early; compromising survival of patients. Due to the rarity of the disease and the heterogeneity of the population, the optimal treatment is still a matter of debate. Surgery remains the mainstay of treatment for localized disease, while radiotherapy and chemotherapy have a role as adjuvant treatments as well as palliative treatments for de novo metastatic or recurrent disease [15]. Despite accumulating data in the field of the role of adjuvant chemotherapy in gynecological sarcomas [17, 20, 26, 27], the significance of this treatment approach is not yet established and its application in clinical practice remains controversial [16–19, 21].

Several prognostic factors have been recognized from retrospective data to guide therapeutic decisions for these patients. Patients with high tumor grade (grade3), advanced stages, high mitotic index and age over 65 are considered to have worse prognosis [4, 5, 7, 28]. In our study, increased mitotic index was the only recognized independent significant prognostic factor in the multivariate analysis. This is in accordance with previous publications in LMS, ESS and UUS [3–5, 29]. Especially for UUS, a recent report by Hardell et al., concluded that UUS should be subdivided to mitogenic and not otherwise specified, according to their mitotic index [30]. Mitotic index was also shown to be of prognostic importance even after neoadjuvant chemotherapy for primary, localized, high grade soft tissue sarcomas [31]. Differences in mitotic index are associated though with different molecular subtypes of the disease that may explain the recognized prognostic significance of the mitotic index in these patients. For example, ESS harboring the YWHAE-FAM22 rearrangement are characterized by significant mitotic activity and clinical aggressiveness in contrast to those associated with JAZF1 rearrangements [32]. Not surprisingly, YWHAE-FAM22 rearrangement in ESS is associated with Cyclin D1 overexpression [30], despite the fact that the mechanism of Cyclin D1 upregulation remains unknown.

Apart from mitotic index, tumor grade was inversely associated with DFS in our analysis. The fact that the grading according to FNCLCC assesses tumor differentiation, mitotic count and necrosis, indicates dependence of grading and mitotic index. This may explain our finding that grade did not retain its significance in the multivariate analysis.

Adjuvant chemotherapy is not the standard of care for uterine sarcomas and several clinical guidelines cannot define its role in the adjuvant setting, even for high-risk to recur patients [15, 22]. Adjuvant chemotherapy in stage I and II leiomyosarcomas failed to prolong the overall survival in a retrospective study of 140 women [16]. Following NCCN and ESMO guidelines about adjuvant chemotherapy in high-risk uterine sarcoma patients, our multidisciplinary team strongly supported the adjuvant approach independently of disease stage but tailoring its administration in each individual case. Thus, only three of the 24 patients included in our study with stage I did not receive adjuvant chemotherapy.

The identification of patients with unfavorable characteristics might be of importance, for both the clinicians and the patients to make the best choice regarding the option of adjuvant chemotherapy. Our analysis identified mitotic index and tumor size > 10 cm as predictors of worse DFS in patients receiving adjuvant chemotherapy. This is in accordance with the prognostic significance of mitotic index. Our study though has the limitation that almost all stage I-III patients included received adjuvant chemotherapy. Therefore, we cannot assess the benefit of adjuvant chemotherapy among specific subgroup of patients. Furthermore, it is noteworthy, that time to progression after 1st line chemotherapy was similar between patients that were de novo metastatic and those recurred after adjuvant chemotherapy. However, no solid conclusion could be drawn for the impact of prior adjuvant treatment since this was a very heterogenous population that received different chemotherapeutic agents.

Molecular drivers and prognostication of uterine sarcomas appear to be evidently different. miRNA profiles of LMS and ESS reveal unique gene signatures [33]. The presence of different fusion genes in low and high grade ESS implies that the molecular pathways involved are distinct [34]. An example of this difference is the high expression of CyclinD1 in ESS harboring the YWHAE/FAM fusion gene, which can also be used as a diagnostic marker and reflects the aggressive behavior of this entity [35]. Mitotic index is an indicator of proliferation which is not used any more for the classification of ESS according to WHO2003 criteria [36]. However, NCCN classification of uterine sarcomas and WHO2014 classification of tumors of the female reproductive organs include mitotic index as a factor that defines tumor grade [22].

## Conclusion

Although, our analysis is limited by its retrospective nature and the relative small number of included patients, due to the rarity of this disease we present real world data for the management of these tumors in a reference center in Greece. Our data are indicative that mitotic index is an important prognostic factor for uterine sarcomas. Mitotic index is an indicator of the aggressive behavior of these tumors that harbor high probability of recurrence independently of disease staging at diagnosis. Despite, our study could not add more clear evidence on the role of adjuvant chemotherapy in these patients, provided further insights on the recognition of baseline factors that affect the prognosis of these rare and aggressive tumors.

## Abbreviations

CIs: Confidence intervals
CT: Computer Tomography
DFS: Disease free survival
ES: Ewing’s sarcoma
ESMO: European Society of Medical Oncology
ESS: Endometrial stromal sarcoma
FIGO: International Federation of Oncology and Obstetrics
FNCLCC: The French Federation of Anticancer Centers
HPF: High power fields
HR: Hazard ratio
LMS: Leiomyosarcoma
NCCN: National Comprehensive Cancer Network
OR: Odds ratios
OS: Overall survival
PFS: Progression free survival
PS: Performance status
RFS: Recurrence free survival
RS: Rhabdomyosarcoma
RT: Radiotherapy
UUS: undifferentiated uterine sarcoma
WHO: World Health Organization
